# Longitudinal reproducibility of brain and spinal cord quantitative MRI biomarkers

**DOI:** 10.1162/imag_a_00409

**Published:** 2025-01-02

**Authors:** Mathieu Boudreau, Agah Karakuzu, Arnaud Boré, Basile Pinsard, Kiril Zelenkovski, Eva Alonso-Ortiz, Julie Boyle, Lune Bellec, Julien Cohen-Adad

**Affiliations:** NeuroPoly, Polytechnique Montreal, Montreal, QC, Canada; Centre de Recherche de l’Institut Universitaire de Gériatrie de Montréal (CRIUGM), Montreal, QC, Canada; Unité de Neuroimagerie Fonctionnelle (UNF), Centre de Recherche de l’Institut Universitaire de Gériatrie de Montréal (CRIUGM), Montreal, QC, Canada; Faculty of Computer Science and Engineering (FINKI), Skopje, Macedonia; Centre de recherche du CHU Sainte-Justine, Université de Montréal, Montreal, QC, Canada; Psychology Department, Université de Montréal, Montreal, QC, Canada; Mila - Quebec AI Institute, Montreal, QC, Canada

**Keywords:** quantitative MRI, longitudinal, reproducibility, biomarker, brain, spinal cord

## Abstract

Quantitative MRI (qMRI) promises better specificity, accuracy, repeatability, and reproducibility relative to its clinically-used qualitative MRI counterpart. Longitudinal reproducibility is particularly important in qMRI. The goal is to reliably quantify tissue properties that may be assessed in longitudinal clinical studies throughout disease progression or during treatment. In this work, we present the initial data release of the quantitative MRI portion of the Courtois project on neural modelling (CNeuroMod), where the brain and cervical spinal cord of six participants were scanned at regular intervals over the course of several years. This first release includes 3 years of data collection and up to 10 sessions per participant using quantitative MRI imaging protocols (T_1_, magnetization transfer (MTR, MTsat), and diffusion). In the brain, T_1_^MP2RAGE^, fractional anisotropy (FA), mean diffusivity (MD), and radial diffusivity (RD) all exhibited high longitudinal reproducibility (intraclass correlation coefficient – ICC ≃ 1 and within-subject coefficient of variations – wCV < 1%). The spinal cord cross-sectional area (CSA) computed using T2w images and T_1_^MTsat^exhibited the best longitudinal reproducibility (ICC ≃ 1 and 0.7 respectively, and wCV 2.4% and 6.9%). Results from this work show the level of longitudinal reproducibility that can be expected from qMRI protocols in the brain and spinal cord in the absence of hardware and software upgrades, and could help in the design of future longitudinal clinical studies.

## 1 Introduction

### 1.1 Quantitative MRI and the reproducibility crisis

Conventional magnetic resonance imaging (MRI) images, commonly used in clinical settings, stem from the application of MRI machines as a non-invasive medical device rather than a scientific instrument (Cercignani et al., 2018;Tofts, 1998). Medical images produced from clinical MRI protocols normally need to be interpreted by expert readers to extract useful diagnostic information, as the images alone typically lack biological specificity and reproducibility due to underlying changes in biology and the electromagnetic fields the imaging hardware generates.

Quantitative MRI (qMRI) techniques (Seiberlich et al., 2020) aim to produce measurements of biological or physical properties through a series of carefully planned conventional MRI images. Quantitative maps are derived from these acquired datasets, which have voxelwise values that typically have physical units associated with them, for example, spin-lattice relaxation time (T_1_[s]), spin-spin relaxation time (T_2_[s]), myelin water fraction (MWF [%]), magnetization transfer ratio (MTR [%]), cerebral blood flow (CBF [ml/g/min]), and diffusion (restricted diffusion coefficients [mm^2^/s], e.g., mean diffusivity (MD) and radial diffusivity (RD)). Some of these measured values are referred to as quantitative imaging biomarkers (QIB) due to their sensitivity to certain biological changes (e.g., myelin loss (Mancini et al., 2020;Schmierer et al., 2007), cerebrovascular diseases and oxygen consumption disorders (Davis et al., 1998;Ma et al., 2016;Mazerolle et al., 2018;Wang et al., 2017), iron deficiency (Lidén et al., 2021;Ropele et al., 2011), etc.). Because these measurements either implicitly or explicitly account for effects that typically are unaccounted for in clinical MRI images, in principle they should have improved reproducibility, which is defined as the reliability of a quantitative imaging biomarker (QIB) in changing conditions that might be expected in a clinical trial or a clinical practice (Kessler et al., 2015;Raunig et al., 2015), such as a longitudinal study.

### 1.2 Longitudinal reproducibility in qMRI: why is it needed?

The longitudinal reproducibility of qMRI biomarkers is an important characteristic to consider when designing longitudinal studies, particularly when clinical features are expected to evolve over time (e.g., worsening disease, or improvement through therapeutic intervention (Oh et al., 2021)). It is also important to know the anticipated variability of these metrics to find the minimum detectable effect size in a power analysis while designing your study. Same-day test-retest studies have shown that fundamental qMRI metrics (e.g., T_1_, T_2_) exhibit low intra-scanner variability in vivo (on the order of 1–2%) (Gracien et al., 2020;Lee et al., 2019). However, such test-retest studies are limited in their usefulness as a longitudinal reproducibility measure because they only consist of two imaging sessions (leading to improper standard deviation calculations) and are done during the same day (same scanner operator, same scanner conditions), which are not realistic conditions experienced during longitudinal studies. It is therefore important to assess longitudinal reproducibility, but can be challenging due to the potential confounds from actual changes of the subject’s tissue properties over time, even from healthy volunteers. Other variables potentially confounding longitudinal reproducibility measures include different scanner operators, magnetic field drifts, and different field shimming. Quantitative MRI metrics in the brain have been shown to correlate with aging in healthy adults (Erramuzpe et al., 2021;Seiler et al., 2020), although changes appear to happen slowly (over decades) and thus short-term longitudinal studies (e.g., 3–5 years) should, in principle, reliably quantify longitudinal reproducibility of a QIB.

### 1.3 Reproducibility in (q)MRI: what’s been done

Many studies have examined reproducibility and repeatability of MRI morphometrics and quantitative MRI. A recent landmark study investigated the longitudinal reproducibility of clinical and functional MRI data of a single subject’s brain acquired on multiple vendors at multiple sites over the course of 15 years (73 sessions across 36 scanners) (Duchesne et al., 2019), finding poor reproducibility across MRI manufacturers for key clinical metrics (i.e., white/grey matter contrast-to-noise ratio (CNR), fluid attenuated inversion recovery (FLAIR) white matter hyperintensities volume). For qMRI metrics, there are several studies that have investigated different aspects of their longitudinal reproducibility. A 7-year scan-rescan brain aging study explored the evolution of quantitative T_1_values in different tissues using the variable flip angle (VFA) technique (which depends on an additional B_1_map) (Gracien et al., 2017) and found T_1_values that were sensitive to aging for this timespan. The reproducibility of quantitative brain metrics when encountering MRI software and hardware upgrades was recently explored in a four time-point, 7-year repeatability and reproducibility study (Salluzzi et al., 2022), which reported the upgrades did not affect the effect size and reproducibility of the tested MRI biomarkers. Reproducibility has also been explored in non-brain anatomy. For spinal cord, between-vendor variability was recently probed by a multi-center (19 sites) study using a generic quantitative MRI spinal cord imaging protocol (Cohen-Adad et al., 2021a) on a single participant over the span of 1 year (Cohen-Adad et al., 2021b). A test-retest quantitative MRI spine study has also been performed in two cohorts (young adult and elderly) over a 10-month period (Lévy et al., 2018), with minimal detectable changes reported for T_1_, magnetization transfer ratio (MTR), magnetization transfer saturation (MTsat), and macromolecular tissue volume (MTV).

### 1.4 Study objective and the CNeuroMod project

The objective of this study was to report the reproducibility of quantitative microstructure MRI metrics across multiple time points in the brain and cervical spinal cord. To do this, two sets of qMRI protocols (brain and spinal cord) were integrated within the Courtois project on neural modeling (CNeuroMod)^*^for collecting longitudinal data on healthy subjects to train and improve artificial intelligence models on brain behavior and activity. The qMRI data acquisition of the brain and spinal cord fell within the “anatomical” imaging branch of the CNeuroMod project, and additional branches of data acquired include deep scanning with functional MRI, biosignals (e.g., cardiac, respiration, eye tracking), and magnetoencephalography (MEG). In addition, we developed reproducible and reusable analysis pipelines for structural qMRI of the brain and spinal cord. These pipelines are built using state-of-the-art tools in terms of pipeline management (Nextflow (Di Tommaso et al., 2017)), structural data analyses (FSL (Smith et al., 2004)), ANTs (Avants et al., 2009), qMRLab (Cabana et al., 2015;Karakuzu et al., 2020), Spinal Cord Toolbox (SCT) (De Leener et al., 2017), and Jupyter notebooks (Beg et al., 2021) with Plotly (Plotly Technologies Inc., 2015) for presenting curated and interactive results in an integrated research object (DuPre et al., 2022), such as a companion Jupyter Book (Executable Books Community, 2020).

## 2 Material and Methods

### 2.1 Data acquisition

**Fig. 1.**
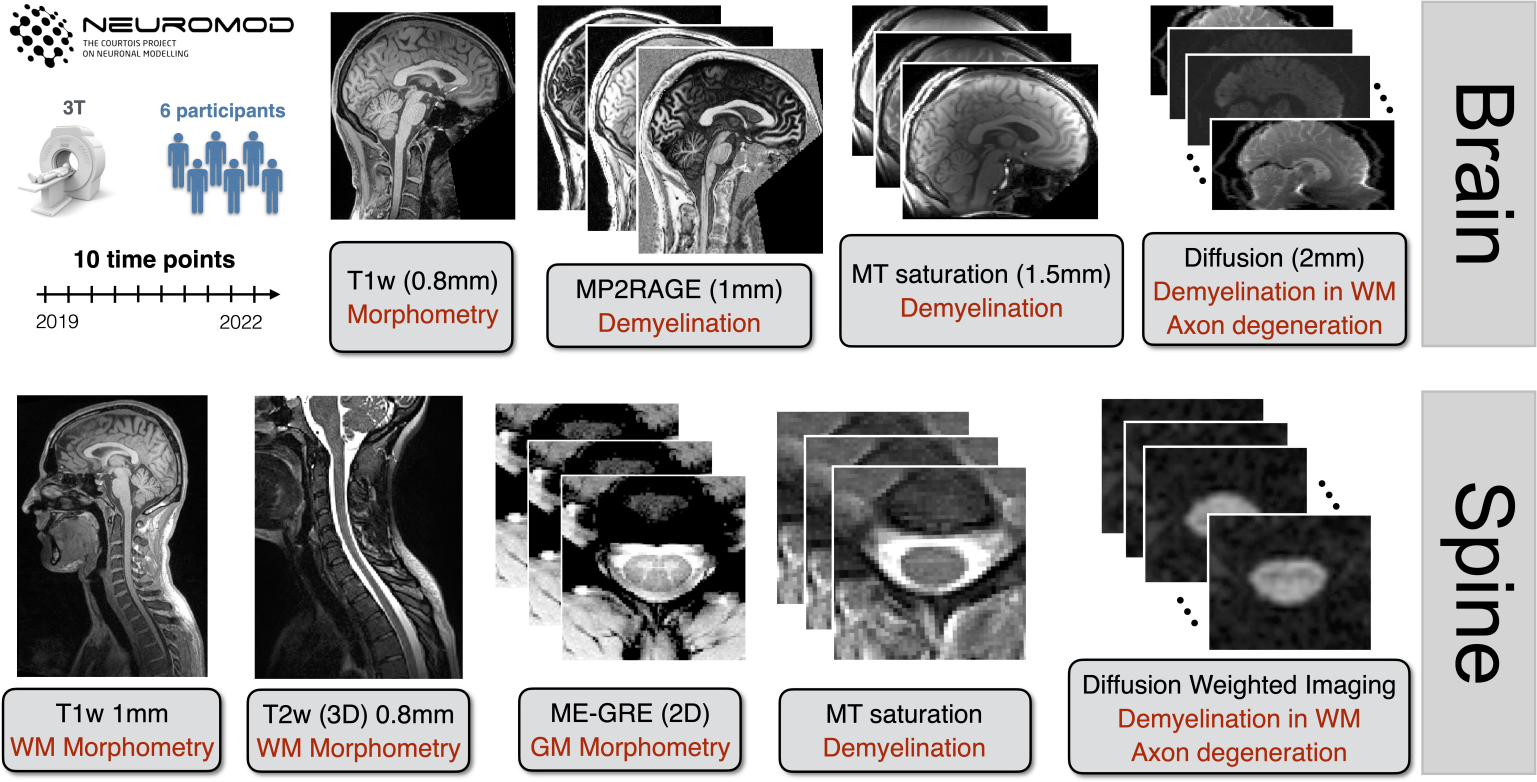
Overview of the structural dataset for the Courtois project on neural modelling (CNeuroMod). Six participants were scanned up to 10 times over 3 years; note that this is an initial data release for 2022. The structural protocol consists of T1w, T2w, and T2*w scans to quantify brain and spinal cord (SC) morphometry. MP2RAGE (Magnetization Prepared 2 Rapid Acquisition Gradient Echoes), magnetization transfer (magnetization transfer ratio, MTR, and magnetization transfer saturation, MTsat), and diffusion-weighted images were used to compute metrics sensitive to demyelination in the white matter (WM).

Six healthy participants (three females) were recruited in 2018 (aged 31 to 47 years old at initial scan date) and consented to be scanned regularly as part of the ongoing Courtois project on neural modelling (CNeuroMod) (J. A. Boyle et al., 2020). All imaging sessions were performed at the same site on a 3.0 T whole-body MRI scanner (Prisma Fit, Siemens, Erlangen, Germany) with a 64-channel head/neck receive coil and 2-channel body transmit coil. The anatomical imaging protocol (Fig. 1) is run on each participant at a rate of approximately four times/year, for 3 years for this initial 2022 data release; more scans are regularly being acquired as the CNeuroMod project is ongoing. Subject number 4 ceased participation after their fifth session. Occasional absences among other participants led to variations in the total number of scans per participant. Each subject had the following number of scans at the time of data processing: subject 1–8 scans, subject 2–10 scans, subject 3–10 scans, subject 4–5 scans, subject 5–8 scans, and subject 6–9 scans. Custom headcases (CaseForge, Berkeley, USA) were used for each participant to minimize motion during the imaging sessions. Although image registration can align images taken over the course of an MRI session, it cannot compensate for the change in static and dynamic magnetic field intensity experienced by the tissue at different head positions. It is crucial for qMRI methods that the intensity patterns of these fields are kept constant for the series of images that are used by a qMRI model (Balbastre et al., 2022;Papp et al., 2016). The same imaging protocol was used for each subject and session. No MRI software or hardware upgrades occurred over the course of the 3-year timespan the data were acquired, and all datasets were acquired over the course of the same 3-year timeframe. Due to the long duration of the study, and the amount of scanning, this project had three different operators running scans, and we also had to vary the time of the exams to accommodate the schedule of participants over several years. Two sets of imaging protocols were implemented, one for the brain and one for the spinal cord, the details of which are summarised next, but are also documented on the CNeuroMod project documentation^†^, including the Siemens MRI exam card PDFs exported from the scanner^‡^.

### 2.2 Brain imaging protocol

The brain imaging protocol (Fig. 1, top) consisted of the following set of acquired MRI images: T1-weighted, T2-weighted, diffusion, MP2RAGE (Magnetization Prepared 2 Rapid Acquisition Gradient Echoes), B_1_mapping, and magnetization transfer (MT) saturation. The T1-weighted image consisted of a 3D MPRAGE acquisition using a repetition time (TR) = 2.4 s, echo time (TE) = 2.2 ms, excitation flip angle (α) = 8 deg, 0.8 mm isotropic resolution, and parallel imaging acceleration factor (R) = 2. The T2-weighted pulse sequence was a 3D fast spin-echo (FSE) acquisition with TR = 3.2 s, TE = 563 ms, 0.8 mm isotropic resolution, and R = 2. The diffusion-weighted protocol was based on the Human Connectome Project (HCP) protocol (Van Essen et al., 2013), and used a 2D axial echo planar imaging (EPI) sequence (TR = 2.3 s, TE = 82 ms, α = 78 deg, 2 mm^3^isotropic resolution, simultaneous multi-slice (SMS) factor of 3, two-shells, 72 directions at 1500 s/mm^2^, 20 directions at 3000 s/mm^2^, and 7 b0 images), and was acquired twice using either P-A or A-P phase-encoding directions, to correct for susceptibility-induced distortion. The MP2RAGE 3D protocol produced two images with different inversion times (TI) = 700 ms and 1500 ms, TR = 4 s, TE = 1.51 ms, α = 7 deg and 5 deg for each TI respectively, 1.2 mm isotropic resolution, and R = 2. B_1_maps were acquired using the default Siemens B_1_mapping sequence based on a gradient echo sequence with ultrafast turbo-FLASH (fast low angle shot) readout (6 mm isotropic resolution) (Chung et al., 2010). Lastly, the MTsat protocol consists of a set of three 3D spoiled gradient echo images: a magnetization transfer-weighted (MTw) image (TR = 28 ms, TE = 3.3 ms, α = 6 deg, 1.5 mm isotropic resolution, R = 2, and a Gaussian-shaped magnetization transfer (MT) preparation pulse with an off-resonance frequency = 1.2 kHz), a proton-density-weighted (PDw) image (same protocol as the MTw, with the omission of the MT preparation pulse), and a T1-weighted (T1w) image (same protocol as the PDw, except TR = 18 ms and α = 20 deg). Note that two different T1w images were acquired with different protocol parameters for different uses: a structural T1w volume for tissue segmentation and registration, and a T1w volume for the MTsat protocol. The total acquisition time for this brain anatomical imaging protocol^§^was 34 min and 47 s.

### 2.3 Spinal cord imaging protocol

The spinal cord imaging protocol (Fig. 1, bottom) consisted of the following set of acquired MRI images: T1-weighted, T2-weighted, T2*-weighted, diffusion, and magnetization transfer (MT) saturation. The T1-weighted image consisted of a 3D MPRAGE acquisition with TR = 2 s, TE = 3.72 ms, α = 9 deg, 1 mm isotropic resolution, and R = 2. The T2-weighted pulse sequence was a 3D fast spin-echo (FSE) acquisition with TR = 1.5 s, TE = 120 ms, α = 120 deg, 0.8 mm isotropic resolution, and R = 3. The T2*-weighted protocol consisted of a multi-echo gradient-echo (ME-GRE) acquisition with TR = 0.6 s, multi-echo combined effective TE = 14 ms, α = 30 deg, 0.9 x 0.9 x 0.5 mm^3^resolution, and R = 2. The diffusion-weighted protocol was based on the Spine Generic protocol (Cohen-Adad et al., 2021a), and used a 2D axial EPI sequence that was cardiac-gated with a pulse oximeter and TR ~ 620 ms, TE = 60 ms, 0.9 mm in-plane resolution, 5 mm slice resolution, phase encoding in the A-P direction, 30 directions at b = 800 s/mm^2^, and 5 b0 images. Lastly, the MTsat protocol consisted of an MTw acquisition (TR = 35 ms, TE = 3.13 ms, α = 9 deg, 0.9 mm^2^in-plane resolution, 0.5 mm slice resolution, R = 2, and a Gaussian-shaped MT preparation pulse with an off-resonance frequency = 1.2 kHz), a proton-density-weighted (PDw) image (same protocol as the MTw, with the omission of the MT preparation pulse), and a T1-weighted (T1w) image (same protocol as the PDw, except TR = 15 ms and α = 15 deg). The total acquisition time for this spinal cord anatomical imaging protocol^**^was 18 min and 22 s.

### 2.4 Data preparation

All datasets acquired within the Courtois project on neural modeling (CNeuroMod) were prepared with the intention to be shared. Data were anonymized and defaced by masking out face, teeth, and ears. Datasets were prepared and organized in the BIDS (Brain Imaging Data Structure) format (Gorgolewski et al., 2016). Quantitative image acquisitions were prepared according to the BEP001 specification (Karakuzu, Appelhoff, et al., 2022), and spinal cord data used the “bp-cspine” tag as proposed in BEP025 to distinguish against the brain datasets for the same subject. Datasets were managed using Datalad (Halchenko et al., 2021) and git-annex in a databank; access to this databank is made available through the CNeuroMod website^††^. Session numbers in the database that are missing for some subjects are omitted datasets from scanning sessions that were aborted due to various scanning issues. sMRIprep (Esteban et al., 2022) was performed on the T1w brain scans from the first two sessions of each subject, which were later published on GitHub using git-annex as part of the CNeuroMod project. These outputs were used solely for the brain diffusion pipeline.

### 2.5 Analysis pipeline

**Fig. 2.**
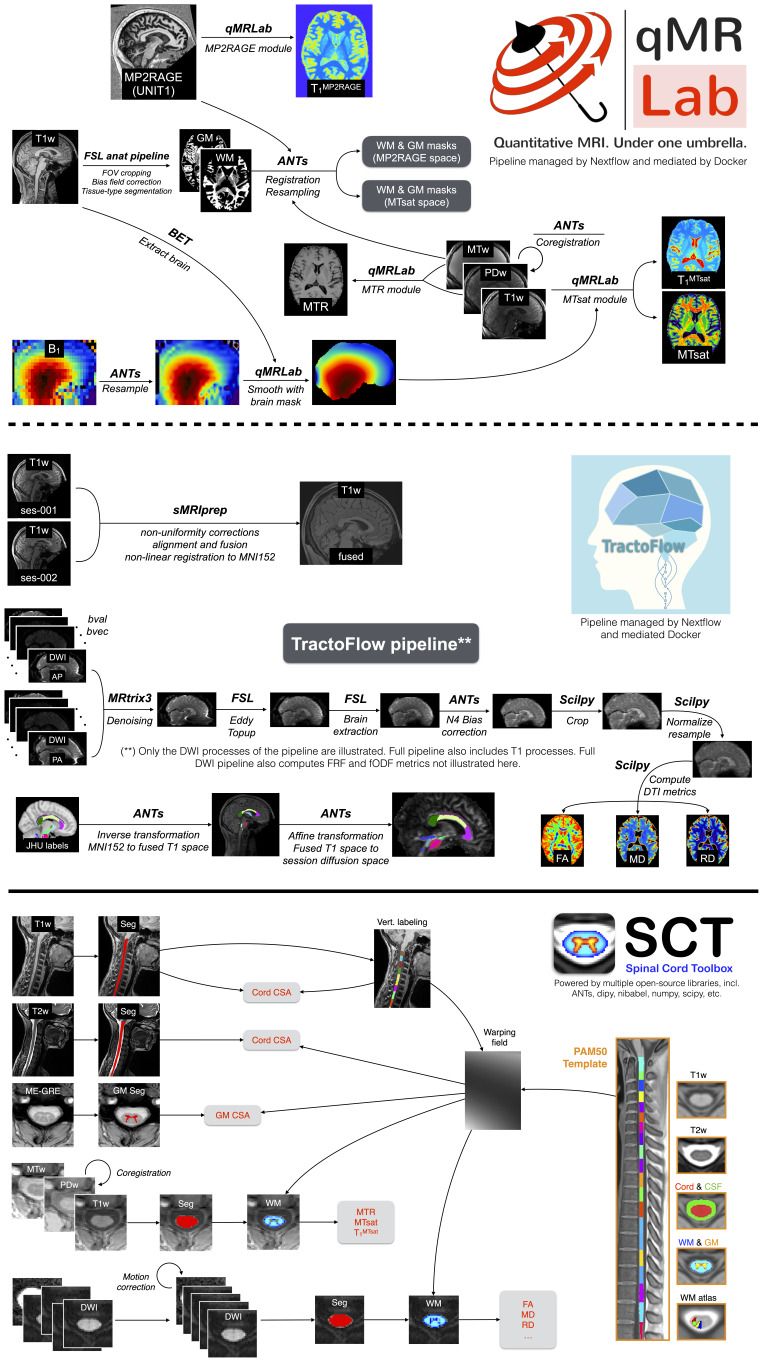
Overview of the three analysis pipelines used in this project: qMRLab (top row), Tractoflow (middle row), and Spinal Cord Toolbox (bottom row). The brain datasets were processed using Nextflow-based pipelines (qMRLab for qMRI processing, and Tractoflow for diffusion processing), whereas spine datasets used a bash script-based pipeline using the Spinal Cord Toolbox software. Several additional software tools were used within these pipelines (e.g., ANTs, FSL, Scipy, MRTrix3, and sMRIPrep). A table listing the input and output files for each automated pipeline can be found in theSupplementary Material B. Note: for both spinal cord and brain imaging, two different T1w images were acquired with different protocol parameters for different uses: a structural T1w volume for tissue segmentation and registration, and a T1w volume for the MTsat protocol.

Two separate post-processing and analysis pipelines were developed for the brain and spinal cord data.Figure 2shows an overview of both pipelines with the outcome metrics.

The brain pipelines were managed using Nextflow (Di Tommaso et al., 2017), a container management tool for data processing pipelines. Two Docker container images were prebuilt and used for this pipeline: dockerhub.io/qmrlab/antsfl:latest (digest: 597de3e6e1aa) and dockerhub.io/qmrlab/minimal:v2.5.0b (digest: 40270330e7b5). Image registration was performed using the Advanced Normalization Tools (ANTS; version 2.1.0) (Avants et al., 2009). Brain extraction was done using the brain extraction tool (BET) tool in the FMRIB Software Library (FSL; version 5.0) (Smith, 2002;Smith et al., 2004), and whole-brain white matter (WM) and grey matter (GM) segmentation were done using the FMRIB’s Automated Segmentation Tool (FAST) in FSL (Zhang et al., 2001). With the exception of diffusion, for all quantitative MRI methods the core data fitting algorithms used in this pipeline are from the open-source qMRLab software (version tag 2.5.0b) (Cabana et al., 2015;Karakuzu et al., 2020). Of note, while the data acquired for the magnetization transfer saturation (MTsat) imaging protocol (Fig. 1) (MTw, PDw, and T1w) generate three output parameter maps (MTsat, magnetization transfer ratio— MTR, and T_1_), only the MTsat parameter maps use all three images in its calculations (equations 7a, 7b, and 7 inHelms et al., 2008,^2010^). MTR is calculated directly from the MTw and PDw images, and T_1_is calculated directly from the PDw and T1w image using equation 7b (Helms et al., 2008,^2010^). For diffusion, the TractoFlow pipeline (version 2.4.1) was used (Theaud et al., 2020), which uses DIPY (Garyfallidis et al., 2014) and MRtrix3 (Tournier et al., 2019) for the core diffusion processing functionalities, and FSL and ANTs for the image processing tools. The diffusion pipeline consists of a denoising step (MRtrix3), TOPUP (using the two phase encoding directions diffusion images) and eddy current corrections (FSL), DTIs (DIPY), brain tissue segmentation (ANTs), and lastly tractography maps (Cousineau et al., 2017); the full processing diagram is shown inFigure 2. DTI metrics were calculated using the 1500 s/mm^2^b-value shell. In addition to the diffusion images as inputs, TractoFlow also used the average of the MPRAGE T1w structural images of the first two sessions (for each subject) that was registered to the MNI152 atlas, which is the output of another standard pipeline, sMRIprep (Esteban et al., 2022), that consists^‡‡^of intensity non-uniformity corrections, alignment and fusion of the images (rigid transformation using FreeSurfer, 6 degrees of freedom), skull stripping, and non-linear registration to the template. The three regions-of-interests (ROIs) of the corpus callosum (genu/body/splenium) were extracted using the John Hopkins University ICBM-DTI-81 WM labels provided by FSL. The labels were first transformed from MNI152 space to the average T1w space (with transformations files available from the sMRIprep outputs^§§^), and then from the average T1w space to the diffusion space using the affine matrix files provided as outputs of TractoFlow.

For the spinal cord data, the pipeline was developed in a shell script^***^using tools all available through the Spinal Cord Toolbox (SCT) v5.6 (De Leener et al., 2017). The script was run through all the available subjects and sessions using the pipeline management tool sct_run_batch. The spinal cord (SC) was segmented on T2w images using sct_deepseg_sc (Gros et al., 2019), and then vertebral levels were automatically identified using sct_label_vertebrae (Ullmann et al., 2014). The SC was then registered to the adult PAM50 template (De Leener et al., 2018). T1w images were analyzed similarly: the SC was segmented, and then the vertebral levels that were estimated on the T2w image were resampled to the T1w image for further computation of cross-sectional area (CSA) within specific levels. Here, we assumed no or minimum motion (few mm) between the acquisition of the T1w and T2w series, which is usually the case (especially since subjects were wearing the CaseForge headcase). It was more reliable to resample the vertebral levels from the T2w image rather than re-estimating them from the T1w image, given that motion artifacts on the T1w image might have caused unreliable vertebral labeling. The ME-GRE images were analyzed using sct_deepseg_gm (Perone et al., 2018) to segment the grey matter. Magnetization transfer (MT) images were processed as follows: The SC was segmented on the MTw scan, followed by registration to the PAM50 template via the T2w-PAM50 transformation. PDw and T1w scans were then registered to the MTw scans. Magnetization transfer ratio (MTR) and magnetization transfer saturation (MTsat) were computed. DWI images were motion-corrected using a mask centred around the SC for more robustness, then registered to the PAM50 template using the initial transformation. DTI metrics were computed using sct_compute_dti (powered by DIPY (Garyfallidis et al., 2014)).The computed metrics are as follows: SC cross sectional area (CSA) averaged between C2-C3 levels from the T1w and T2w scans (using sct_process_segmentation), GM CSA averaged between C3-C4 from the ME-GRE scan, MTR, MTsat, T_1_and DTI metrics extracted in the WM between levels C2-C5.

For both head and spine quantitative MRI data, the mean value was first computed over the region of interest (i.e., segmented white matter or grey matter for the outputs of the qMRLab and SCT pipelines, and three regions of the corpus callosum for the brain diffusion maps— genu, body, and splenium). To evaluate the longitudinal reproducibility of the measured metrics in a test-retest scenario, a within-subject coefficient of variation was computed (wCV) for each subject. For wCV, the coefficient of variation (wCV = σ_w_/x¯_w_, where σ is the standard deviation of the measured values and x¯ is the mean) across all sessions was computed for a given quantitative MRI metric, and then the mean of this CV was calculated across all subjects. To estimate the variation of the measured metrics between subjects, a between-subject CV was computed (bCV). For bCV, the mean value of the quantitative metric across all sessions was computed for each subject, and then the CV (σ_b_/x¯_G_, where x¯_G_is the grand mean) of these means was computed across all subjects. Note, bCV is not a measure of the accuracy of the qMRI measurements, but instead may provide information on how sensitive the metric is to biological differences between healthy subjects. Lastly, the intraclass correlation coefficient (ICC), widely accepted as an aggregate measure of reproducibility and repeatability (Raunig et al., 2015), was computed (Equation 1) for each metric and ROI,

$$ICC=\frac{\sigma_{b}^{2}}{\sigma_{b}^{2}+\sigma_{w}^{2}}$$ (1)

where$\sigma_{w}^{2}$is the mean variance for each subject for a given QIB, and$\sigma_{b}^{2}$is the variance of the mean values of that QIB between subjects. This formula is equivalent to the ICC (1) designation in (McGraw & Wong, 1996). A high ICC (~1) paired with a low within-subject variance ($\sigma_{w}^{2}$) or low within-subject CV (wCV = σ_w_/x¯_w_) is thus an indicator of high reproducibility of a QIB measurement for that study interval and for a sample size that is sufficiently representative of the population.

### 2.6 Quality control (QC)

For brain qMRI data processing (excluding diffusion), quality assurance was done manually with the assistance of the Nextflow log, which provides a report on success/failure of each processing step for all subjects and sessions. The resulting maps and masks were also visually verified manually, which resulted in some subsequent corrections to how the tissue masks were calculated^†††^. A slab profile effect in magnetization transfer saturation (MTsat) data due to improper protocol planning was discovered during QC^‡‡‡^, and this effect was not present in the B_1_maps. In response to this discovery, it was decided to remove the top 20 and bottom 20 slices of the volume from the WM and GM masks. The imaging volume varied slightly between sessions as it was controlled by the scanner operator (for example, the head position of subject 1 varied in the superior-inferior direction by a standard deviation of 3.75 voxels), thus so that some of the volume that was masked out. Although many slices of the*volume*were removed (40 out of 96), there was only a small reduction in total WM and GM voxels counted for the “whole-brain” metrics (for example, for subject 1, an average of 8 ± 2% of WM voxels and 11 ± 1% of GM voxels were removed from the volume). Five data points from the brain quantitative MRI pipeline (Fig. 2, top) were omitted due to missing B_1_maps in the Courtois project on neural modeling (CNeuroMod) database at the time of processing for these subject’s sessions: sub-03_ses-003, sub-06_ses-001, sub-06_ses-002, sub-06_ses-003, sub-06_ses-005. For these sessions, the data points from the brain diffusion pipeline (Fig. 2, middle) and spine pipeline (Fig. 2, bottom) were not removed as these pipelines do not require B_1_as an input.

For brain diffusion data processing, a report was generated from the TractoFlow tool*dmriqc_flow*(v 0.2.0— (Theaud & Descoteaux, 2022)). Each step of the pipeline has been manually validated without any reported issues. Two sessions were excluded due to corrupted initial acquisitions (sub-03_ses-002, sub-03_ses-003). For the spinal cord data processing pipeline, a QC report showing various steps of the analysis (segmentation, vertebral labeling, registration) was generated and made publicly available on the GitHub project repository (https://github.com/courtois-neuromod/anat-processing, release version r20220804). Following expert readings, some data points were excluded due to factors such as excessive motion (sub-05_ses-007 [T2w]), poor shimming (sub-03_ses-010 [T1w] and sub-05_ses-007 [T1w]), and incorrect volume placement or incorrect b-values (sub-02_ses-001 [DWI], sub-03_ses-003 [DWI], sub-06_ses-008): details are listed in GitHub issues (https://github.com/courtois-neuromod/anat-processing/issues). In addition, the pipeline failed to produce an output for two data points (sub-04_ses-001, sub-06_ses-005). A table with the listed included and excluded sessions for each subject is shared as Supplementary Material (Supplementary Material A).

## 3 Results

### 3.1 Brain

Average quantitative MRI (excluding diffusion) values for the segmented whole-brain^§§§^white matter (WM) and grey matter (GM) for each subject and session are shown inFigure 3. Missing data points are either unacquired sessions or because they were excluded after doing quality control. Note that the magnetization transfer ratio (MTR) is calculated from a subset of the images acquired in the magnetization transfer saturation (MTsat) imaging protocol, and B_1_is not shown because it is only used as a transmit radiofrequency (RF) field correction factor for the MTsat calculation, and does not have biological specificity.

**Fig. 3.**
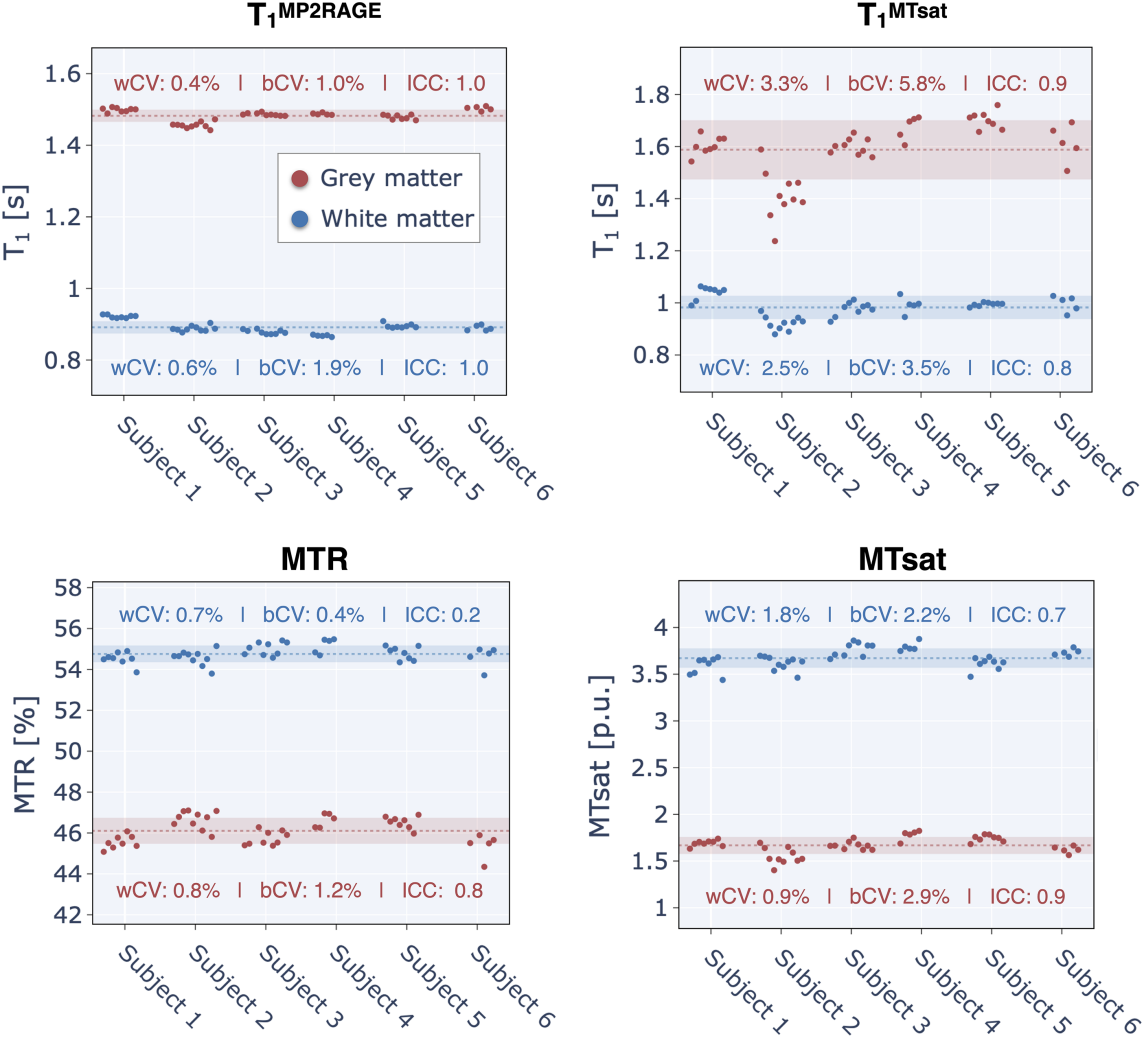
Brain qMRI metrics (excluding diffusion). Each point represents the mean metric within the white matter (WM) or grey matter (GM) for one subject and one session. Missing data points are due to unacquired sessions, the pipelines failing to produce an output, or were excluded due to quality control (See Quality Control section for more details). The brain volume segmented for MTsat data was slightly smaller than the MP2RAGE data volume (for MTsat, this reduction amounted to 8% for WM and 11% for GM) due to a slab-profile effect, as discussed in the Quality Assurance section. The dotted line represents the mean across all subjects and sessions, while the shaded region illustrates the range of values within one standard deviation (SD) of the mean (mean ± SD). The within- and between-subject coefficient of variations (wCV and bCV) for these metrics in WM and GM are shown inside each respective plot. Note: subject 4 stopped participating after their fifth session.

T_1_values estimated from MP2RAGE demonstrated the highest longitudinal reproducibility for this set of QIBs in WM and GM, with an intraclass correlation coefficient (ICC) approximately equal to 1 and a within-subject coefficient of variation (wCV) of less than 1%. In comparison, T_1_values derived from the MTsat dataset exhibited a similar ICC (WM: 0.7, GM: 0.8) but higher wCV (WM: 2.5%, GM: 3.3%). These findings indicate good reproducibility relative to the two other metrics derived from the MTsat dataset (MTR and MTsat). MTR and MTsat had lower ICC values but smaller wCV values than for T_1_^MTsat^, meaning that although the measurements had good test-retest precisions in all but MTR in WM, the between-subject coefficient of variation (bCV) was low. This indicates that these two QIB were less sensitive to between-subject differences than for T_1_in this healthy cohort. MTR in WM had a higher bCV than wCV, meaning the within-subject QIB variability was higher over this timespan than the biological variability of the QIB for this population sample, suggesting MTR has lower biological sensitivity than the other three QIBs.

**Fig. 4.**
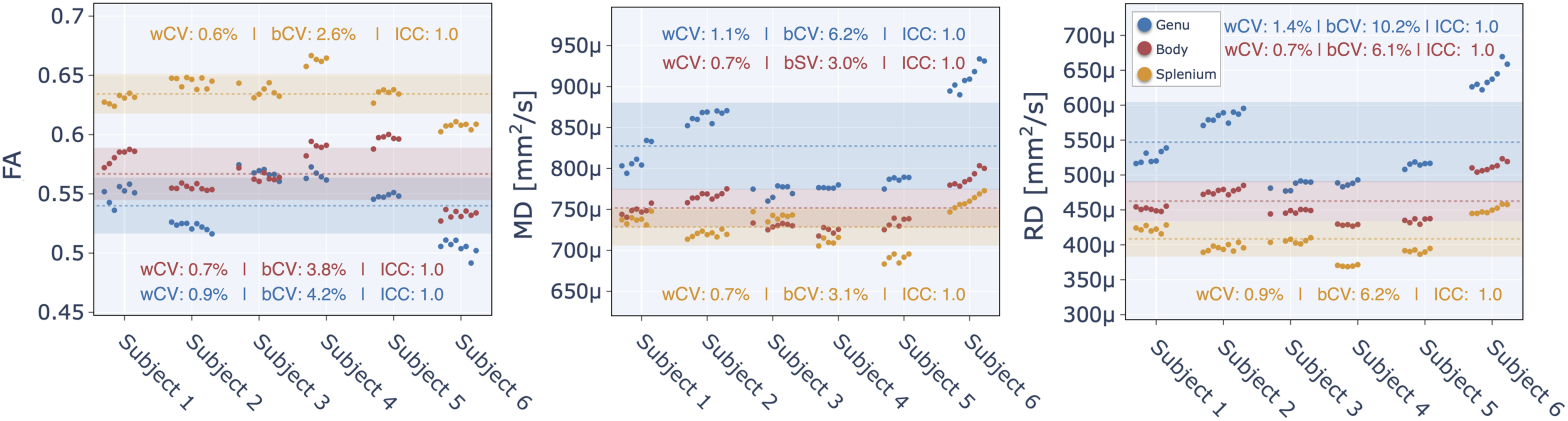
The mean diffusion metrics (fractional anisotropy – FA, mean diffusivity – MD, and radial diffusivity – RD) for each acquired session are shown for three atlas-based regions of the corpus callosum (genu in blue, body in red, splenium in yellow) of each subject. The dotted line represents the mean across all subjects and sessions, while the shaded region illustrates the range of values within one standard deviation (SD) of the mean (mean ± SD).

Figure 4displays the three calculated diffusion QIBs (fractional anisotropy — FA, mean diffusivity — MD, and radial diffusivity — RD) within the main corpus callosum regions (genu, body, splenium). All three QIB exhibited very high longitudinal repeatability (ICC ≃ 1 and wCV$\underset{˜}{<}$1%). These QIBs also demonstrated excellent sensitivity for biological differences in this population sample; as in addition to high ICC, they nearly all had bCV values that exceeded 3%, implying that these ROIs in each subject had diffusion QIB values which are specific to each subject and were relatively stable (as can be seen inFig. 4). The lowest wCV value is reported for fractional anisotropy (FA) in the body (0.6%), and it also had the lowest bCV value (2.6%).

### 3.2 Spinal cord

**Fig. 5.**
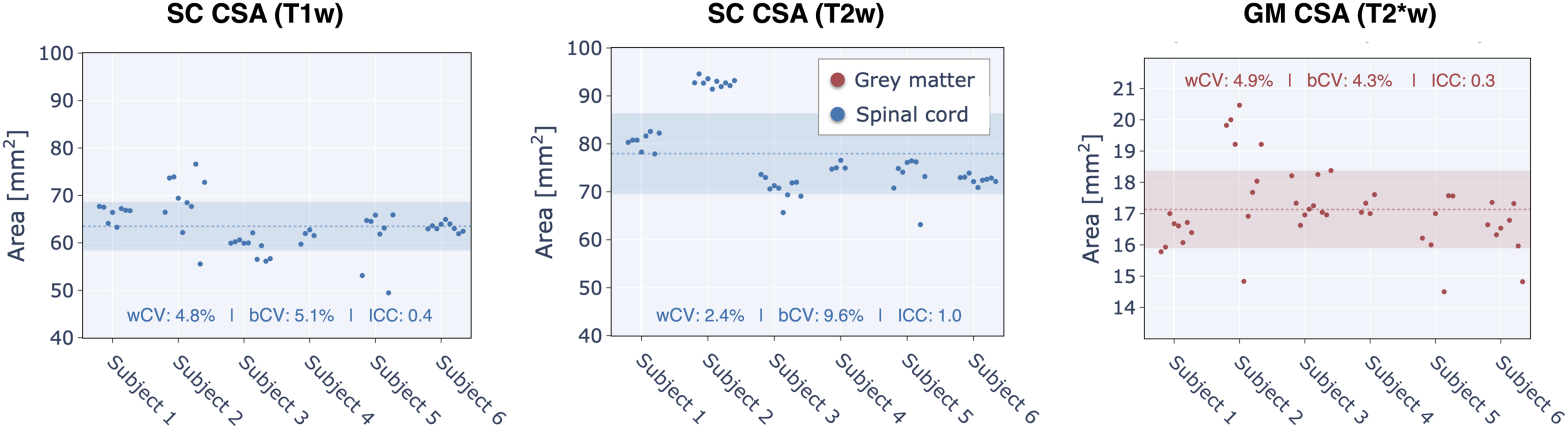
Spinal cord cross-sectional area (CSA) for each acquired subject and session (using either the T1w or T2w images) and in grey matter (GM) (using the T2*w images). The dotted line represents the mean across all subjects and sessions, while the shaded region illustrates the range of values within one standard deviation (SD) of the mean (mean ± SD).

Figure 5displays the results for the spinal cord (SC) cross-sectional area (CSA) calculated for SC (using T1w and T2w images) and grey matter (GM) (using T2*w images). SC CSA across the C2 and C3 vertebral levels calculated with T2w images resulted in the best longitudinal reproducibility for the CSA QIB in WM (intraclass correlation coefficient— ICC ≃ 1 and within-subject coefficient of variation— wCV = 2.4%). SC CSA computed using T1w images had low longitudinal reproducibility due to wCV ≃ bCV (ICC = 0.4), and wCV for T1w (4.8%) was also twice the value of wCV for T2w SC CSA (2.4%). GM CSA values computed from T2*w images had the lowest intraclass correlation coefficient (ICC = 0.3), with a high wCV (4.9%) and wCV > bCV. We observed a particularly high bCV for SC CSA computed using T1w for subject 2, which was due to subject motion between scans for some sessions and resulted in unreliable spinal cord segmentation. In addition, the overall higher wCV for SC CSA calculated from T1w images versus T2w images is likely due to a difference in image volume position; for T1w, the C2-C3 slices were at the anterior edge of the imaging volume, which in some instance resulted in lower segmentation quality than for the T2w C2-C3 slices, which were located closer to the center of the imaging volume. To avoid rater bias in the within- and between-subject statistics, the analysis pipeline was fully automated, and no mask was manually edited.

**Fig. 6.**
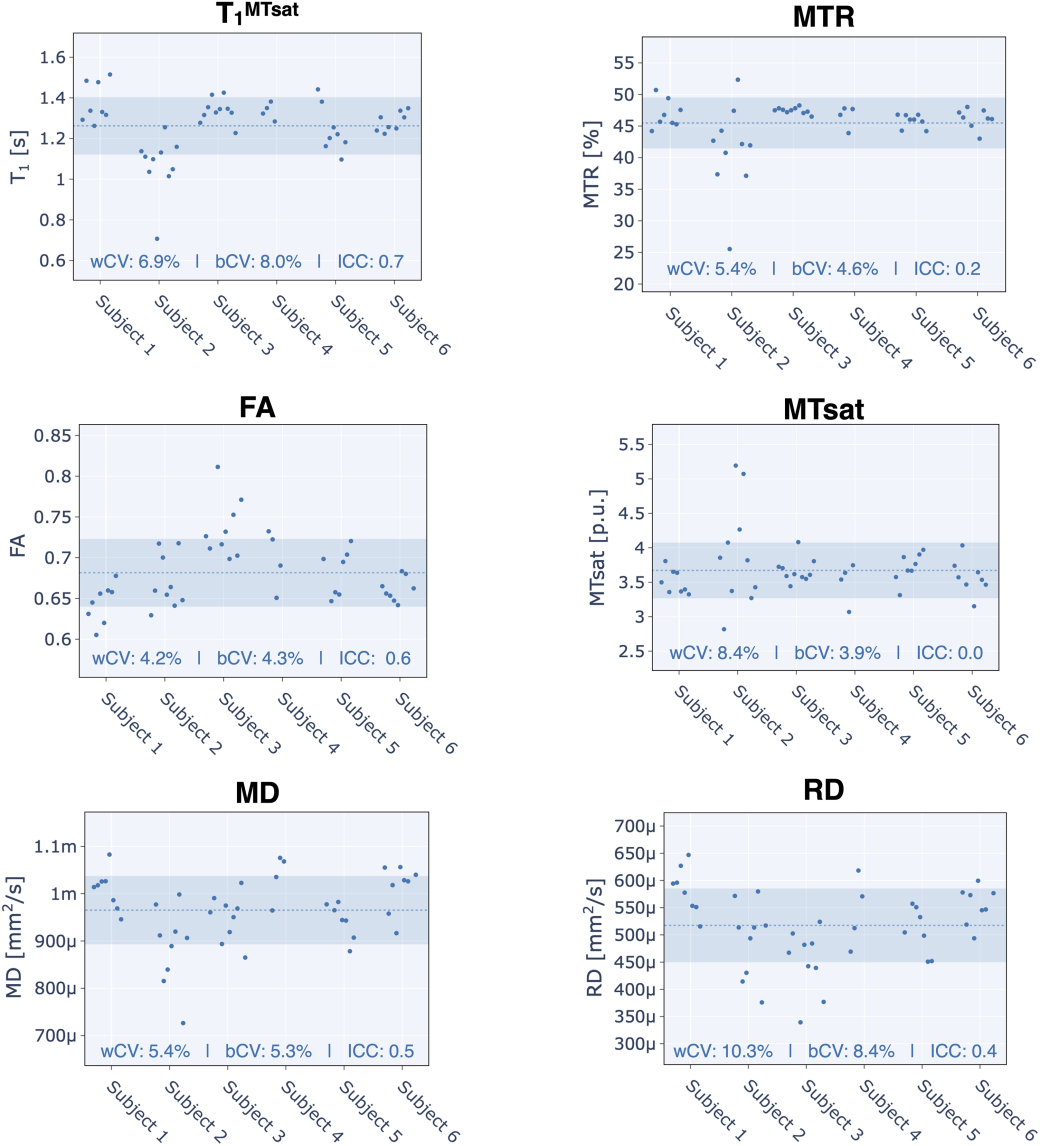
Spinal cord qMRI metrics (T_1_, magnetization transfer ratio — MTR, magnetization transfer saturation— MTsat, fractional anisotropy — FA, mean diffusivity — MD, radial diffusivity — RD). Each point represents the mean metric within the white matter (WM) across C2 and C5 levels, for one subject and one session. The dotted line represents the mean across all subjects and sessions, while the shaded region illustrates the range of values within one standard deviation (SD) of the mean (mean ± SD).

Figure 6shows the scatter plots of all qMRI QIB mean values calculated in the WM across the C2 and C5 vertebral levels of the spinal cord. For this set of QIB in the spinal cord, T_1_^MTsat^exhibited the best longitudinal reproducibility (ICC ≃ 0.7 and wCV = 6.9%). The diffusion QIBs exhibited modest reproducibility over this timespan (ICC ≃ 0.5 and wCV ≃ bCV). The magnetization transfer ratio (MTR) and magnetization transfer saturation (MTsat) QIBs had the lowest reproducibility (ICC ≃ 0, high wCV values ≃ 10%, and wCV > bCV).

## 4 Discussion

Longitudinal reproducibility of quantitative MRI metrics is an important feature for clinical and research studies that intend to use the MRI scanner as a scientific instrument. In this study, we examine the longitudinal reproducibility of a fundamental MR parameter (T_1_) and microstructural biomarkers (magnetization transfer ratio – MTR, magnetization transfer saturation – MTsat, diffusion) within the central nervous system (brain and spinal cord) over a 3-year period at a single imaging site. We report high intraclass correlation coefficient (ICC) paired with low within-subject coefficient of variation (wCV) values that indicate good reproducibility for the T_1_, MTsat, FA, MD, and RD QIBs in the brain. The qMRI metrics that exhibited the lowest reproducibility was the magnetization transfer ratio (MTR) in WM, exhibiting a low ICC (0.2) and wCV ≃ bCV (between-subject coefficient of variation). A potential reason for the better reproducibility of T_1_estimates from MP2RAGE versus MTsat is that the MP2RAGE acquisition protocol is inherently optimized to reduce sensitivity of B_1_effects (Marques et al., 2010), whereas T_1_measured from MTsat has high sensitivity to B_1_errors (Boudreau et al., 2017,^2018^) and thus requires an additional B_1_map to calibrate the nominal excitation flip angles. Inaccuracies in the B_1_maps will propagate as errors in the T_1_maps even with this calibration step, which may impact reproducibility measures. The use of an inversion pulse in MP2RAGE may induce more magnetization transfer effect than the MTsat pulse sequence. Another factor contributing to higher wCV and bCV values of the MTsat/MTR/T_1_^MTsat^is the removal of slices needed to avoid the slab observed profile effect (as discussed in the Quality Control section) and thus these data do not accurately represent whole-brain data (for subject one, it contained 92 ± 2% of WM voxels and 89 ± 1% of GM voxels). In addition, the head positions varied slightly between sessions (for subject 1, the standard deviation of this variation was 3.75 voxels), and the percentage of whole-brain data also varied slightly between sessions.

Spinal cord cross-sectional area (CSA) had wCV values of 4.8% and 2.4% for those calculated from T1w and T2w scans, respectively. The almost two-fold smaller wCV for CSA computed on the T2w scan is likely due to the higher robustness to subject motion and/or spinal cord pulsatile motion for the T2w fast spin echo sequence versus the T1w MPRAGE. This is consistent with a recent study (Bautin & Cohen-Adad, 2021), where wCV values were 0.8% for T1w images and 0.57% for T2w images. Note that theBautin and Cohen-Adad (2021)study was based on in-silico generation of scan-rescan using random affine transformations, hence the variability was highly under-estimated compared to the present study. In our study, the reported CVs are likely closer to a realistic longitudinal scenario and suggest good long-term repeatability for this quantitative metric in the spinal cord, and that T2w is the better choice for SC CSA measurements. Our CV values are consistent with another related multi-site and multi-manufacturer study (Cohen-Adad et al., 2021b), where one subject was scanned in 19 different imaging centers over a period of 77 days, and also 42 groups scanned 6 different subjects at their imaging sites resulting in 260 datasets. Our reported longitudinal CV values for MTR and MTsat (5.4% and 8.4%) are on the order of Cohen-Adad et al. within-site CVs (3.6% and 11%). These overall agreements between a multi-center snapshot in time and a single-center longitudinal study provide encouraging evidence for the longitudinal reproducibility when imaging the spinal cord.

Our SC wCV values are overall higher than for the brain. When going through the SC QC report, we noticed issues with some diffusion scans, for example, manifesting as signal dropout sporadically across time (likely due to patient motion), or systematically across all time points and across multiple slices (likely caused by wrong positioning of the imaging FOV and/or 2DRF excitation volume). This reinforces the need for a standard operating procedure when imaging the spinal cord (Cohen-Adad et al., 2021a).

Although the mean measured T_1_values from MP2RAGE and MTsat across our population sample were consistently different, with a mean difference of 91 ms in WM and 105 ms in GM across repeated measurements over 3 years, their values are within the range reported in the literature for WM at 3T (690–1100 ms) (Stikov et al., 2015). Additionally, the 95% limits of agreement, ranging from 11 to 171 ms in WM and 99 to 309 ms in GM, highlight the variability between these measurement methods and underscore that caution should be taken when comparing T_1_values measured with different techniques in longitudinal studies. The average T_1_value we calculated using MTsat data in the spinal cord WM was also high (1263 ms) relative to literature MP2RAGE T_1_values in the spinal cord (920 ms, (Baucher et al., 2021)). The reason for this overestimation is not known, but may be explained in part by the omission of a B_1_map in the Spine Generic Protocol (Cohen-Adad et al., 2021a) to calibrate the flip angles in the MTsat equations. Thus, adding B_1_mapping to the Spine Generic Protocol should be explored in future iterations to ensure better accuracy. Improving the accuracy of these quantitative MRI techniques is beyond the scope of our work, which is being explored by others through the development of quantitative MRI phantoms (Golay & Oliver-Taylor, 2022;Stupic et al., 2021), multi-site studies using a standardized phantom (Bane et al., 2018;Boudreau et al., 2024;Keenan et al., 2021), and vendor-neutral pulse sequences (Karakuzu, Biswas, et al., 2022).

Another longitudinal study (Salluzzi et al., 2022) investigated the short-term repeatability and long-term reproducibility in a healthy cohort over a 5 year interval with a different set of quantitative MRI metrics (T_2_/T_2_^*^, quantitative susceptibility, cerebral blood flow, and diffusivity). Their work, though investigating mostly different metrics, is complementary to our study in that its main objective was to assess the potential impacts of both software and hardware MRI upgrades on the repeatability and reproducibility of this set of qMRI metrics. They reported wCV values on the order of 1% or less for diffusion metrics (FA/MD/RD) in the three corpus callosum regions, in agreement with the observations reported in our study. Lastly, the range of FA/MD/RD values we reported in the corpus callosum (FA: 0.49–0.67, MD: 683–934 10-4 mm^2^/s, RD: 369–670 10-4 mm^2^/s) is similar to those reported by Salluzzi et al. (FA: 0.48–0.70, MD: 806–1334 10-4 mm^2^/s, RD: 407–1000 10-4 mm^2^/s), and is also similar to those reported for the 30- to 50 year-old range in healthy subjects in a recent aging study (Pietrasik et al., 2020).

### 4.1 Limitations

Some limitations related to this study are important to highlight. Foremost, all MRI acquisitions in this work were done on a single MRI scanner, and thus a single MRI vendor. MRIs from different vendors could have varying reproducibility and repeatability performance due to their hardware components and proprietary pulse sequences. To evaluate these limitations to our own study, future work could explore multi-vendor reproducibility and repeatability using both the proprietary pulse sequence and open-source pulse sequence frameworks. There has been a lot of recent work on open-source pulse sequence frameworks (Cordes et al., 2020;Karakuzu, Biswas, et al., 2022;Layton et al., 2017) aiming to minimize these differences and give more control to the user researchers that may provide a solution to this limitation. Alternatively, between-vendor biases can be accounted for in the statistics analysis step (Hagiwara et al., 2019), or by using a standard system phantom (Keenan et al., 2021).

We reported on the longitudinal reproducibility of mostly coarse regions-of-interest in the brain and spinal cord (whole-brain white matter (WM) and grey matter (GM) mean values, in-plane WM and GM spinal cord means), except for the brain diffusion metrics which were averaged for the three corpus callosum regions as was similarly done in (Salluzzi et al., 2022). More granular masking methods exist for both the brain and spinal cord (e.g., white & grey matter (Desikan et al., 2006;Lévy et al., 2015;Oishi et al., 2009), tractometry (Catani & Thiebaut de Schotten, 2008)), and may be explored in the future. Also, due to our small population sample size (n = 6), bCV may not properly reflect the true value for the population. Future studies using these methods should consider more subjects.

Long-term biological changes in brain tissue occur naturally in healthy individuals due to macro- and microstructural effects associated with normal aging (MacDonald & Pike, 2021). Given that our study spanned 3 years and focused solely on adults in mid-adulthood (aged 31 to 47 years old at the initial scan date), we anticipate that the natural effects of aging on the brain, such as myelin generation/degradation and ventricular enlargement, would manifest slowly during this period (Ge et al., 2002;Hagiwara et al., 2021;Steen et al., 1995).

Our study used a readily available B_1_mapping sequence provided by the MRI manufacturer to balance scan efficiency and participant comfort. Although it is practical to use readily available sequences, more recent B_1_mapping methods may have better accuracy and reproducibility (Boudreau et al., 2017). Because actual B_1_fields are known to be smoothly varying (Sled & Pike, 1998;Sled et al., 1998), we followed the recommended practice of filtering the B_1_maps to remove noise or small image artefacts (Boudreau et al., 2017); however, a limitation of the filtering process is that it does risk introducing some errors in neighboring voxels.

In general, motion during MRI scans and the repositioning of participants across scanning sessions can contribute to lower longitudinal reproducibility. This work is part of a larger study, CNeuroMod, whose main goal was to generate functional MRI data of the highest possible quality, over 5 years. To minimize motion during scans, the CNeuroMod study implemented two specific measures. First, custom headcases produced by CaseForge were used to reduce motion. These headcases have been shown to effectively decrease motion levels; however, this reduction is comparable to widely used approaches such as applying medical tape for tactile feedback or using cushions (Jolly et al., 2020;Power et al., 2019). The headcases also helped maintain consistent head positioning within the head coil throughout the study, potentially increasing measurement consistency compared to more typical longitudinal acquisitions. Longitudinal studies that do not employ dedicated motion-reduction techniques may experience lower reproducibility of quantitative MRI (qMRI) metrics than reported here.

Second, the CNeuroMod study recruited participants who were unusually committed to data quality, agreeing to weekly scanning sessions over 5 years. This high level of scanning experience and commitment may have resulted in lower motion levels compared to typical subjects, although motion levels have been shown to be, in general, a relatively stable and heritable trait (Achterberg & van der Meulen, 2019). We did not collect motion measures during anatomical scans to directly test this hypothesis. However, the fMRI scanning sessions provided extensive estimates of motion during various tasks which showed low-to-moderate motion levels with two participants (sub-03 and sub-04) exhibiting unusually low motion (J. Boyle et al., 2023). Overall, the motion levels in our sample represent a realistic range but do not feature participants with high motion, which may not reflect the diversity of participants observed, notably, in clinical populations.

The processing pipelines were all only automatic, and no manual interventions were done during the segmentation steps of the pipeline. Manual corrections or more robust tools would likely improve the reliability of the reported metrics in both brain and spinal cord. Our open-source pipelines had low failure rates, and the failures were due to missing data for specific sessions (e.g., missing B_1_map). As our pipelines are fully automated and were designed for this specific study, external users may experience a higher failure if scanning conditions change substantially (e.g., field strength, anatomy, pulse sequence parameters, etc.). However, like any pipeline, researchers should not apply the pipeline as is, but rather try to understand its design so it can be adapted for the purpose of their data. For example, if researchers acquire data with an excessive amount of noise in the DWI spinal cord scans, then the motion correction step would likely need to be revisited, for example, by grouping more subsequent DWI volumes to increase the robustness of co-registration. There are hundreds of parameters in processing pipelines, and researchers should know them and their impact, before applying a pipeline to their data. Although outside of the scope of this current study, the reproducibility of quantitative morphometry in the brain (e.g., cortical thickness) could also be explored and compared against the quantitative MRI metrics using this open dataset.

## 5 Conclusion

Our study assessed the longitudinal reproducibility of several MRI quantitative imaging biomarkers (T_1_, MTR, MTsat, FA, MD, RD, and SC CSA) in the brain and cervical spinal cord over 3 years at a single site. We report a high intraclass correlation coefficient and low within-subject coefficient of variation for most of the QIBs, indicating good longitudinal reproducibility. Future work would benefit from an increased sample size to get a better estimate of the between-subject variability, particularly for QIBs that had high within-subject variability.

The results of this initial data release, which can be made available upon request, may be used as a benchmark for the development of other analytical methods. Additionally, this study forms a component of the larger ongoing project, CNeuroMod. The long-term quantitative MRI database may provide valuable information for integration in deep learning training models with data acquired using other techniques (e.g., fMRI, MEG) that may account for confounding changes in the subjects’ brains.

## Supplementary Material

Supplementary Material

## Data Availability

An interactive NeuroLibre preprint of this manuscript (doi: 10.55458/neurolibre.00018) is available athttps://preprint.neurolibre.org/10.55458/neurolibre.00018/. In the aim of better reproducibility and transparency in research, all the data, processing pipelines, containers, and analysis code have been made available online. The anonymized and defaced datasets are in BIDS format and managed using Datalad and git-annex in a GitHub repository,https://github.com/courtois-neuromod/anat(commit: 5a5f687), and the data itself are hosted on a self-hosted S3 server. The sMRIPrep pipeline outputs for each subjects are also managed using git-annex and GitHub,https://github.com/courtois-neuromod/anat.smriprep(commit: b055f52). To request access to this data, we invite researchers to fill out an application form on our websitehttps://www.cneuromod.ca/access/access/. The brain quantitative MRI processing pipeline was written in Nextflow (brain) and shell (spine) and is available in this repository:https://github.com/courtois-neuromod/anat-processing. The TractoFlow pipeline is built using open-source tools and is available on GitHub:https://github.com/scilus/tractoflowcombined with the container image on Dockerhub: dockerhub.io/scilus/scilus:1.4.2 (digest: 25415e45ea7f,https://hub.docker.com/repository/docker/scilus/scilus). The qMRI brain pipeline used two Docker containers which have been made available as saved container images on Dockerhub: dockerhub.io/qmrlab/antsfl:latest (digest: 597de3e6e1aa,https://hub.docker.com/repository/docker/qmrlab/antsfsl) and dockerhub.io/qmrlab/minimal:v2.5.0b (digest: 40270330e7b5,https://hub.docker.com/repository/docker/qmrlab/minimal). The condensed outputs of these pipelines (e.g., masked and averaged values for each tissue) are shared in GitHub releases of this repository, which can be found here:https://github.com/courtois-neuromod/anat-processing/releases/. The data figures and tables in this article were produced using analysis code integrated in an interactive Jupyter Book and powered by Plotly, which is available here,https://courtois-neuromod.github.io/anat-processing-paper/, and the code repository for this book ishttps://github.com/courtois-neuromod/anat-processing-paper.

## Footnotes

*
https://www.cneuromod.ca/

†
https://docs.cneuromod.ca/en/latest/MRI.html#brain-anatomical-sequences

‡
https://docs.cneuromod.ca/en/latest/_downloads/4f1f285d300373cc60a45d4085097bad/anatomical_protocol_2019-01-22.pdf

§
https://docs.cneuromod.ca/en/latest/MRI.html#brain-anatomical-sequences

**
https://docs.cneuromod.ca/en/latest/MRI.html#spinal-cord-anatomical-sequences

††
https://www.cneuromod.ca/access/access/

‡‡ The pipeline diagram for the external tool sMRIprep is available in their documentation:https://www.nipreps.org/smriprep/

§§
https://github.com/courtois-neuromod/anat.smriprep.git

***
https://github.com/courtois-neuromod/anat-processing/blob/main/process_spinalcord.sh

††† Release r20220916:https://github.com/courtois-neuromod/anat-processing/releases/tag/r20220916

‡‡‡ Release r20220921:https://github.com/courtois-neuromod/anat-processing/releases/tag/r20220921

§§§ For MTsat data, a small section of the superior and anterior of the brains were removed due to a slab-profile effect. See the Quality Assurance section for a discussion and the extent of this reduction in terms of “whole-brain” values.
