# Supplementary material for: Longitudinal reproducibility of brain and spinal cord quantitative MRI biomarkers
Source: Imaging Neurosci (Camb). 2025 Jan 2;3:imag_a_00409. doi: 10.1162/imag_a_00409 (PMC12319937; doi:10.1162/imag_a_00409)
Supplement: Supplementary Material [file imag_a_00409-supp.pdf]

Subject	Session	qMRLab	SCT	Tractoflow	Diffusion protocol
1	1	Data point plotted	Data point plotted	Data point plotted	Original
1	2				Original
1	3				Original
1	4				Original
1	5				Original
1	6				Original
1	7				Original
1	8			Skipped due to different DWI protocol	New
1	9	GitAnnex - Not available Sept 2022	GitAnnex - Not available Sept 2022	Skipped due to different DWI protocol	New
1	10	GitAnnex - Not available Sept 2022	GitAnnex - Not available Sept 2022	Skipped due to different DWI protocol	New
2	1				Original
2	2				Original
2	3				Original
2	4				Original
2	5				Original
2	6				Original
2	7				Original
2	8				Original
2	9				Original
2	10			Skipped due to different DWI protocol	New
3	1				Original
3	2			Tractoflow no output (reason unknown): missing f	Original
3	3	qMRLab pipeline no output (reason: missing B1 map*)		Tractoflow no output (reason unknown): missing f	Original
3	4				Original
3	5				Original
3	6				Original
3	7				Original
3	8				Original
3	9				Original
3	10			Skipped due to different DWI protocol	New
4	1		**SCT pipeline fail - no output (unknown reason)**		Original
4	2				Original
4	3				Original
4	4				Original
4	5				Original
4	6	Subject stopped participating	Subject stopped participating	Subject stopped participating	New
4	7				New
4	8				New
4	9				New
4	10				New
5	1				Original
5	2				Original
5	3				Original
5	4				Original
5	5				Original
5	6				Original
5	7			Skipped due to different DWI protocol	New
5	8			Skipped due to different DWI protocol	New
5	9	GitAnnex - Not available Sept 2022	GitAnnex - Not available Sept 2022	Skipped due to different DWI protocol	New
5	10	Not acquired yet (March 1 2023)	Not acquired yet (March 1 2023)	Not acquired yet (March 1 2023)	
6	1	qMRLab pipeline no output (reason: missing B1 map)			Original
6	2	qMRLab pipeline no output (reason: missing B1 map)			Original
6	3	qMRLab pipeline no output (reason: missing B1 map)			Original
6	4				Original
6	5	qMRLab pipeline no output (reason: missing B1 map)	**SCT pipeline fail - output, but 0 and null (unknown reason)**		Original
6	6				Original
6	7				Original
6	8				Original
6	9			Skipped due to different DWI protocol	New
6	10	GitAnnex - Not available Sept 2022	GitAnnex - Not available Sept 2022	Skipped due to different DWI protocol	New
		*Note: I had notified through a GitHub issue of the missing B1 maps ("famp" suffix files), but the issue was later closed without actually uploading the missing data.			
		**Note: Only datasets included in the first data release were included in the analysis of this publication. Subsequent releases (e.g. March 1 2023 data release) were not included.			

Supplementary Material B. Table of inputs and outputs for each automated pipeline. Full pipeline diagram is shown in Figure 2.

**qMRLab**	• B1 map<br>• T1w image<br>• MTsat dataset<br>• MP2RAGE dataset	• Filtered & resampled B1 map<br>• Two sets of WM & GM binary masks (resampled and registered to the MTsat and MP2RAGE datasets)<br>• T1 map (from the MP2RAGE dataset)<br>• T1/MTR/MTsat maps (from MTsat and B1 datasets)
**TractoFlow**	• Two T1w images (from different sessions<br>• Two DWI dataset (AP and PA phase-encoding directions)<br>• John Hopkins University ICBM-DTI-81 WM labels	• A fused T1w dataset<br>• FA map<br>• MD map<br>• RD map<br>• AD (axial diffusivity) map (unused)
**SCT**	• T1w image<br>• T2w image<br>• ME-GRE dataset<br>• DWI dataset<br>• MTsat dataset	• Vertebral label mask<br>• T1/MTR/MTsat maps (from MTsat dataset)<br>• FA map<br>• MD map<br>• RD map<br>• CSV files for each metric (SC CSA T1w, SC CSA T2w, GM CSA T2*, FA, MD, RD, T1, MTR, and MTsat) with statistics (mean, STD) across their respective vertebral range for that dataset.

Supplementary Material C.

Image registration details and parameters used for all pipelines.

qMRLab pipeline

For the qMRLab NextFlow pipeline, all registration was done via ANTS (“antsRegistration” command), and all the parameters are stored in the NextFlow configuration file: https://github.com/courtois-neuromod/anat-processing/blob/36281fecc15f7f9aa7a17f19b7713e84617002f4/nextflow.config#L134-L143

For convenience, here they are:

```
ants_dim=3
ants_metric="MI"
ants_metric_weight=1
ants_metric_bins=32
ants_metric_sampling="Regular"
ants_metric_samplingprct=0.25
ants_transform="Rigid[0.1]"
ants_convergence="[1000x500x250x100,1e-6,10]"
ants_shrink="8x4x2x1"
ants_smoothing = "3x2x1x0vox"
```

It uses a rigid transformation (6 degrees of freedom) with gradient steps 0.1.

SpinalCordToolbox pipeline

For the SpinalCordToolbox pipeline (https://github.com/courtois-neuromod/anat-processing/blob/main/process_spinalcord.sh), registration is done using the SpinalCordToolbox with either the command “sct_register_to_template” (to register an image to the PAM50 template) or “sct_register_multimodal” command (to register one acquired image with another). For both commands, the registration parameter options are provided via the “-param” argument. Here are few examples for each of the ones above from our pipeline:

—

https://github.com/courtois-neuromod/anat-processing/blob/36281fecc15f7f9aa7a17f19b7713e84617002f4/process_spinalcord.sh#L161-L162

```
# Register to PAM50 template

sct_register_to_template -i ${file_t2}.nii.gz -s ${file_t2_seg}.nii.gz -l ${file_label}.nii.gz -c t2 -param step=1,type=seg,algo=centermassrot:step=2,type=seg,algo=syn,slicewise=1,smooth=0,iter=5:step=3,type=im,algo=syn,slicewise=1,smooth=0,iter=3 -qc ${PATH_QC} -qc-subject ${SUBJECT}
```

—

https://github.com/courtois-neuromod/anat-processing/blob/36281fecc15f7f9aa7a17f19b7713e84617002f4/process_spinalcord.sh#L202-L204

```
# Register PD->T1w
# Tips: here we only use rigid transformation because both images have very similar sequence parameters. We don't want to use SyN/BSplineSyN to avoid introducing spurious deformations.

sct_register_multimodal -i ${file_mtoff}.nii.gz -d ${file_t1ax}.nii.gz -dseg ${file_t1ax_seg}.nii.gz -param step=1,type=im,algo=rigid,slicewise=1,metric=CC -x spline -qc ${PATH_QC} -qc-subject ${SUBJECT}
```

—-

https://github.com/courtois-neuromod/anat-processing/blob/36281fecc15f7f9aa7a17f19b7713e84617002f4/process_spinalcord.sh#L213-L214

```
# Register template->T1w_ax (using template-T1w as initial transformation)

sct_register_multimodal -i $SCT_DIR/data/PAM50/template/PAM50_t1.nii.gz -iseg $SCT_DIR/data/PAM50/template/PAM50_cord.nii.gz -d ${file_t1ax}.nii.gz -dseg ${file_t1ax_seg}.nii.gz -param
```

```
step=1,type=seg,algo=slicereg,metric=MeanSquares,smooth=2:step=2,type=
im,algo=syn,metric=CC,iter=5,gradStep=0.5 -initwarp
warp_template2T2w.nii.gz -initwarpinv warp_T2w2template.nii.gz -qc
${PATH_QC} -qc-subject ${SUBJECT}
```

—

As can be seen above, the registration parameters are shared between some steps, but can differ for others, depending on the source and reference images being used. Here are the definitions for each unique algorithms used in our pipeline, taken from the SpinalCordToolbox documentation (https://spinalcordtoolbox.com/user_section/tutorials/registration-to-template/template-registration/customizing-registration.html#customizing-registration-section):

algo=rigid:
Axial (X-Y) translation + rotation about the Z axis.

algo=slicereg:
Slice-by-slice axial (X-Y) translation, regularized along the Z axis. This can be used as both an initial alignment of a segmented cord centerline, or to align the centers of two images that are already close.

algo=centermassrot:
An alignment of the center of mass of the segmented cord with the center of mass of the template. Similar to algo=slicereg, but also includes rotation, to account for a turned cord due to e.g. compression on one side of the cord.

For all of these listed algorithms, SpinalCordToolbox calls ANTS commands (specifically, antsRegistration) to register the images.

TractoFlow pipeline

For the TractoFlow pipeline, there are two steps using registration: a registration step done prior to the TractoFlow Nextflow call to prepare the T1w image, and then within the TractoFlow/Nextflow.

sMRIprep preprocessing

The first one is done via sMRIprep. This pipeline uses a wrapper for FreeSurfer's https://surfer.nmr.mgh.harvard.edu/fswiki/mri_robust_template to fuse the set of T1w images, using 6 degrees of freedom to robustly register the image using https://surfer.nmr.mgh.harvard.edu/fswiki/mri_robust_register. So, unlike the previous, this step uses FreeSurfer for image registration instead of ANTs. More preprocessing is done, but here is the main registration call: https://github.com/nipreps/smriprep/blob/45c2e2fc088839979f18b83be630a6f9d515b416/smriprep/workflows/anatomical.py#L1489-L1502

Following this, the fused image is registered non-linearly with the `MNI152NLin2009cAsym` brain template, https://github.com/nipreps/smriprep/blob/45c2e2fc088839979f18b83be630a6f9d515b416/smriprep/workflows/fit/registration.py#L95, which is done using antsRegistration, https://github.com/nipy/nipype/blob/d2f4953f964cd07a791bea2eadc94f4af1aa3f81/nipype/interfaces/ants/registration.py#L628. The processing loops a set of custom antsRegistration settings in order to find the optimal nonlinear registration of the fused brain to the template, and the configuration files for this set of settings are listed here:

- https://github.com/nipreps/niworkflows/blob/dc783d88f89f0baf36f36bde035c588d0db91d14/niworkflows/data/t1w-mni_registration_fast_000.json
- https://github.com/nipreps/niworkflows/blob/dc783d88f89f0baf36f36bde035c588d0db91d14/niworkflows/data/t1w-mni_registration_precise_000.json
- https://github.com/nipreps/niworkflows/blob/dc783d88f89f0baf36f36bde035c588d0db91d14/niworkflows/data/t1w-mni_registration_precise_001.json
- https://github.com/nipreps/niworkflows/blob/dc783d88f89f0baf36f36bde035c588d0db91d14/niworkflows/data/t1w-mni_registration_precise_002.json

For convenience, here is one of these configuration files (t1w-mni_registration_precise_000.json):

```
{
  "collapse_output_transforms": true,
  "convergence_threshold": [0.000001, 0.000001, 0.000001],
  "convergence_window_size": [20, 20, 10],
  "dimension": 3,
  "interpolation": "LanczosWindowedSinc",
  "metric": ["Mattes", "Mattes", "CC"],
  "metric_weight": [1, 1, 1],
  "number_of_iterations": [
    [100, 100],
```

```
    [100, 100],
    [100, 70, 50, 20]
  ],
  "output_transform_prefix": "ants_t1_to_mni",
  "output_warped_image": true,
  "radius_or_number_of_bins": [56, 56, 4],
  "sampling_percentage": [0.25, 0.25, 1.0],
  "sampling_strategy": ["Regular", "Regular", "None"],
  "shrink_factors": [
    [2, 1],
    [2, 1],
    [
      8,
      4,2, 1]
  ],
  "sigma_units": ["vox", "vox", "vox"],
  "smoothing_sigmas": [
    [2, 1],
    [1, 0],
    [
      3,
      2,1, 0]
  ],
  "transform_parameters": [
    [0.05],
    [0.08],
    [0.1, 3.0, 0.0]
  ],
  "transforms": ["Rigid", "Affine", "SyN"],
  "use_histogram_matching": [true, true, true],
  "winsorize_lower_quantile": 0.005,
  "winsorize_upper_quantile": 0.995,
  "write_composite_transform": true
}
```

Main TractoFlow pipeline

As for TractoFlow itself, it uses ANTs registration tools to do so during the “TOPUP” correction,

https://github.com/nipreps/smriprep/blob/45c2e2fc088839979f18b83be630a6f9d515b416/smriprep/workflows/anatomical.py#L1489-L1502

```
antsRegistrationSyNQuick.sh -d 3 -f $b0 -m ${sid}__rev_b0_mean.nii.gz
-o output -t r -e 1
```

(which, note that it uses antsRegistrationSyNQuick instead of simply antsRegistration, see: https://github.com/ANTsX/ANTs/blob/master/Scripts/antsRegistrationSyNQuick.sh for details about the input argument descriptions)

And then for registration against the fused T1w image provided by sMRIPrep:

https://github.com/scilus/tractoflow/blob/7d69625136fbdb9e32fddbd23997f88d144e374c/main.nf#L1422-L1439

```
antsRegistration --dimensionality 3 --float 0\
        --output
[output,outputWarped.nii.gz,outputInverseWarped.nii.gz]\
        --interpolation Linear --use-histogram-matching 0\
        --winsorize-image-intensities [0.005,0.995]\
        --initial-moving-transform [$b0,$t1,1]\
        --transform Rigid['0.2']\
        --metric MI[$b0,$t1,1,32,Regular,0.25]\
        --convergence [500x250x125x50,1e-6,10] --shrink-factors
8x4x2x1\
        --smoothing-sigmas 3x2x1x0\
        --transform Affine['0.2']\
        --metric MI[$b0,$t1,1,32,Regular,0.25]\
        --convergence [500x250x125x50,1e-6,10] --shrink-factors
8x4x2x1\
        --smoothing-sigmas 3x2x1x0\
        --transform SyN[0.1,3,0]\
        --metric MI[$b0,$t1,1,32]\
        --metric CC[$fa,$t1,1,4]\
        --convergence [50x25x10,1e-6,10] --shrink-factors 4x2x1\
        --smoothing-sigmas 3x2x1
```
